# A Comprehensive Review of Experimental Rodent Models of Repeated Blast TBI

**DOI:** 10.3389/fneur.2019.01015

**Published:** 2019-09-27

**Authors:** Maciej Skotak, Molly T. Townsend, Kakulavarapu V. Ramarao, Namas Chandra

**Affiliations:** Department of Biomedical Engineering, Center for Injury Biomechanics, Materials, and Medicine, New Jersey Institute of Technology, Newark, NJ, United States

**Keywords:** low-level blast, repeated blast exposure, peak overpressure, inter-exposure interval, Sprague-Dawley rat, Long-Evans rat, C57BL/6J mouse

## Abstract

We reviewed the relevant literature delineating advances in the development of the experimental models of repeated blast TBI (rbTBI). It appears this subject is a relatively unexplored area considering the first work published in 2007 and the bulk of peer-reviewed papers was published post-2011. There are merely 34 papers published to date utilizing rodent rbTBI models. We performed an analysis and extracted basic parameters to capture the characteristics of the exposure conditions (the blast intensity, inter-exposure interval and the number of exposures), the age and weight of the animal models most commonly used in the studies, and their endpoints. Our analysis revealed three strains of rodents are predominantly used: Sprague Dawley and Long Evans rats and wild type (C57BL/6J) mice, and young adult animals 8 to 12-week-old are a preferred choice. Typical exposure conditions are the following: (1) peak overpressure in the 27–145 kPa (4–21 psi) range, (2) number of exposures: 2 (13.9%), 3 (63.9%), 5 (16.7%), or 12 (5.6%) with a single exposure used for a baseline comparison in 41.24% of the studies. Two inter-exposure interval durations were used: (1) short (1–30 min.) and (2) extended (24 h) between consecutive shock wave exposures. The experiments included characterization of repeated blast exposure effects on auditory, ocular and neurological function, with a focus on brain etiology in most of the published work. We present an overview of major histopathological findings, which are supplemented by studies implementing MRI (DTI) and behavioral changes after rbTBI in the acute (1–7 days post-injury), subacute (7–14 days), and chronic (>14 days) phases post-injury.

## Introduction

The research on combat injuries is traditionally invigorated during and in the aftermath of military conflicts which increases the likelihood to sustain combat-related trauma among warfighters. The complexity and severity of these injuries vary and have been covered extensively by numerous retrospective studies published over the years (1–3). The review of Rustemeyer et al. (2) identified combat injury patterns which characterize combat operations using 10 studies selected from the period 1982 to 2005. They found differences in the causality: fragmentation injuries were predominant during the 90s compared to the Vietnam War, where shooting injuries were the most common. The trunk injuries were reduced in conflicts from 1991 onwards with the advances in military personal protective equipment (PPE) including combat body armor (CBA). They also concluded the mortality of wounded soldiers in all conflicts was consistently between 10 and 14%, and these numbers were even lower for the Afghanistan and Iraq operational theaters (4, 5). The review of Tong et al. concluded that the side effect of the deployment of CBA was an increase in combat-related head, face, and neck (HFN) injuries among service personnel wearing CBA in Iraq and Afghanistan (6). Modern ceramic plate CBA has decreased the incidence of fatal-penetrating injuries to the torso but offers no protection to the limbs and face which remain exposed to gunshot wounds and fragments from improvised explosive devices (IEDs). Three major contributing factors were identified: the increased survivability of soldiers because of CBA, fragmentation injuries from explosive devices, and the lack of protection to the face and limbs. The higher incidence of injuries caused by fragmentation to the HFN region was attributed to the more common use of IEDs and other explosive devices. The military helmet offers protection against ballistic projectiles and fragmentation, but studies from our laboratory and by the others (7–9) suggest that Advanced Combat Helmet (ACH) and derivatives offer limited to no protection against the blast waves. It is one of the leading root causes behind the epidemics of the blast-related mild TBI in veterans (10, 11). Of all soldiers returning from Iraq and Afghanistan with blast-induced mild TBI, 43.9% of them have been shown to have PTSD (11) and considerable effort has been devoted toward discriminating the symptoms of blast mTBI and PTSD. It was concluded that battlefield PTSD is most likely to be a psychological consequence of physical trauma and it is solely caused by exposure to an over-pressure wave that is generated by the blast (12).

Despite extensive human studies, until relatively recently the most mystifying among the combat injuries was undoubtedly Blast-Induced Neurotrauma (BINT). The efforts using animal models helped formulate the body of knowledge and shed light on the effects of the blast wave on the central nervous system (CNS). In the early days of animal model research on BINT was focused on demonstration whether a shock wave alone can cause measurable physical injury to the brain structures, and subsequently can lead to long term pathological and behavioral changes. A few excellent reviews have been published on the subject, and the informed reviewer is directed to these works for a detailed summary of the findings (13–15). These animal studies used an educated guess approach with respect to the exposure conditions, considering there is no publicly available data on the exposure conditions in theater. Even in case studies where there was no doubt the subject was exposed to multiple blast waves terms like “large blast” were used to describe the intensity of the blast waves (16). Similarly, the early attempts at neurological effects in training with heavy weapons or breaching lack characterization of exposure conditions (17, 18). Only in recent years, the deployment of the B3 blast gauges held the promise to acquire the exposure parameters (19, 20), which can be used as injury predictors, if correlated with the observed neurological abnormalities. The service life of the blast gauge in the combat zone was relatively short (2011–2016) and ended with a decision to abandon the program (21). There is growing concern about the neurological effects of exposure to low-level blast (LLB) experienced by military and law enforcement members during the training (22). One of the advantages of the training setting compared to the active combat zone is ordered sequence of events, predetermined beforehand, which facilitates precise monitoring of the individual and cumulative overpressure dosages. These dosages can be then correlated with blood biomarker levels, and results of neurocognitive tests applied timely. The characteristics of the overpressure dosage (the most critical set include peak overpressure, impulse, and inter-exposure interval, Δt) can be easily obtained from the blast gauges worn by trainees. One successful attempt to extract these vital data is presented in the paper by our group working in collaboration with the Walter Reed Army Institute of Research (WRAIR) team lead by Dr. Kamimori (23). The significance of the availability of this kind of data is paramount: for the first time, it creates an opportunity to develop animal models based on quantified human data. In our view, this is a unique opportunity in the TBI research area where human exposure data are usually not available.

This review focuses on the scrutiny of existing models of repeated blast TBI with an emphasis on rodent models using shock tubes. We performed the analysis to identify existing exposure paradigms, strains of animal models (age, body mass) used, the focus areas of the studies and we summarize the most important findings. The guidelines for the future development of animal models of LLB are also included.

## Repeated Blast TBI Rodent Models: An Overview of the Experimental Variables

**Figure 1:**
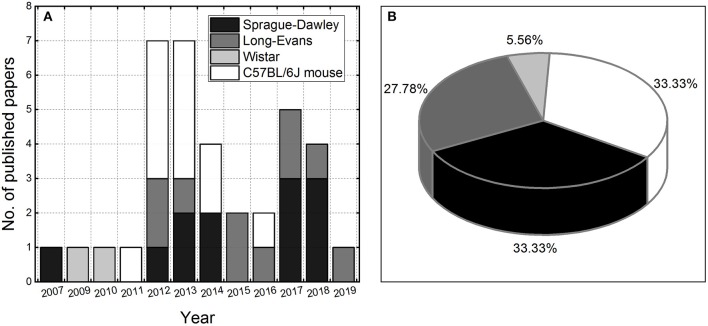
The number of peer-reviewed papers published per year on repeated blast TBI using rodent models **(A)**. The pie chart illustrating the utilization of rodent models in rbTBI studies included in the analysis **(B)**.

This review is focused on the presentation of advances made in the development of experimental animal models of repeated blast TBI. The literature review indicates it is a relatively new and unexplored research area, considering the number of papers (36) published in the last 12 years (Figure 1). Three strains of rodents are the most frequently used for this purpose: C57BL/CJ mouse (12 papers) and Sprague-Dawley (12 articles) and Long-Evans rats (10 publications), with two papers which used Wistar rats (24, 25). We extracted basic exposure parameters used in these studies: peak overpressure, number of exposures, and the interval between consecutive exposures, to gain an insight on existing practices and evaluate their relevance for the future development of the animal models of low-level blast exposure.

Two animal related basic parameters typically reported are body mass and the age at injury. Surprisingly the age of rodents used in the studies was reported only in 55.6% of the papers (20 out of 36), the body mass was reported in 88.9% of the articles (32 out of 36), while both parameters were reported in 50% of the time. The age of the mice was usually 8 to 10 weeks (11 out of 12 papers), and only in one instance, they were older (12–16 weeks, reported in all 12 articles) (26). The age of Long-Evans rats was 10–12 weeks (4 out of 10 articles), and Sprague-Dawley rats 8–10 weeks (3 out of 12 articles).

The typical body mass of the mice was in the 21–26 grams range (reported in 10 out of 12 papers). Not surprisingly, the reported body mass variation was much higher among the rats (min.-max.): 250–400 g (Long-Evans, reported in all 10 published papers) and 200–400 g (Sprague-Dawley, reported in 10 out of 12 papers). It would appear that these two strain have different growth rates with a clear body mass difference at 8 weeks onwards [see Figure 1 in Turner and Burne (27)], and for LE rats the housing conditions (standard vs. enriched environment) might also play a role. At 16 weeks the body mass disparity almost reached the threshold of statistical significance [*t*_(10.85)_, *p* = 0.052] between standard and enriched environment housed animals (27). A comparison of the growth rates of a Sprague-Dawley rats form two different sources, Charles River Labs (CRL) and Harlan Laboratories (HAR) lead to conclusions that animals from CRL were gaining weight at a more rapid rates compared to HAR rats starting at 10 weeks of age (28). However, when the second cohort of animals from both sources was subjected to a similar study the overall findings from the first cohort were confirmed, but there was obvious difference in growth rates (body mass) between cohorts from the initial time point (6–7 weeks). The analysis of weekly food intake per 100 g body weight revealed some disparities at 17 and 19 weeks, but overall there were no differences. Body composition, organ weights, tibial lengths and blood parameters were measured. CRL rats showed higher body fat mass (49.6%), liver weights (22.2%), lower testicular weights (30.8%), and lower cholesterol levels (25.4%) than HAR rats. This study demonstrated that the outbred SD rats obtained from two different sources showed significant differences in growth rates which were manifested differences in body composition most likely caused by differences in genetic makeup. Authors also cautioned that “the difference in body composition among various sources will be a potential confounding factor in model creation and its effects on therapeutic intervention, thus making the model and therapy less reliable (28).”

**Figure 2:**
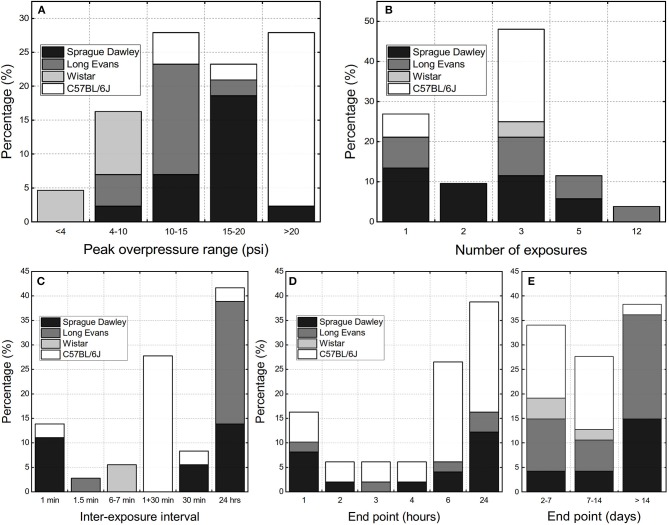
The overview of the experimental design parameters used in the repeated bTBI studies with rodent models expressed as percentage of published peer-reviewed papers: **(A)** peak overpressure ranges, **(B)** number of exposures, **(C)** inter-exposure interval. The terminal endpoints used in the studies: **(D)** within 24 h post-injury, **(E)** classified as “acute” (<7 days), “sub-acute” (7 to 14 days), and “chronic” (>14 days).

We performed the analysis of the experimental variables reported in available rbTBI literature using as inclusion criteria only studies conducted in shock tubes and where repeated exposure was performed on rodent models. These criteria yielded 36 peer-reviewed papers. There are three main parameters evaluated: peak overpressure, number of exposures, and inter-exposure interval, which was also supplemented by analysis of experimental endpoints (Figure 2). Majority of studies used relatively high blast overpressures (BOPs): in 80% of reported data blast intensities higher than 69 kPa (10 psi) were used (Figure 1A). Three blast exposures were the most popular injury paradigm (66% of the cases, Figure 2B) and as well as the Δt of 24 h between consecutive injuries (42% of the data, Figure 2C). The focus of most of the studies were acute pathological changes with the endpoint of 24 h the most frequently used (38%, Figure 2D). The analysis of the distribution of the endpoints with a 7-day bin size indicates there is a balance between “acute” (<7 days), “subacute” (7–14 days) and “chronic” characterization (inset in Figure 2D). These results indicate that animal models developed to replicate occupational low-level blast conditions a fine-tuning of experimental variables is necessary. Notably, peak overpressures of <70 kPa (10 psi) should be employed, the number of exposures increased, which should be accompanied by a shortening of the inter-exposure intervals.

### Long-Evans Rats

Studies on effects of repeated blast exposure which employed Long Evans rats almost exclusively used Δt of 24 h, relatively low BOPs, generally below 75 kPa, and the number of repetitions which between 3, 5, and 12 (Table 1). Majority of the studies focused on chronic effects including comprehensive characterization of behavioral changes, with less emphasis on the acute characterization.

**Table 1:** The summary of experimental variables (exposure conditions: BOP, number of exposures and inter-exposure interval, analyzed brain region, tissue or organs, behavioral testing and end points) in studies using Long Evans rats.

**BOP, kPa**	**Number of exposures**	**Inter-exposure interval**	**Brain region (other tissue or organ)^**a**^**	**Behavior**	**End points**^**b**^			**References**
					**Acute, hours**	**Sub-acute, days**	**Chronic, days**	
75	3	24 h	C, H, WB				42, 98, 224, 280	(29)
75	3	24 h	H	+			175	(30)
69	1, 5	24 h	TG, Plasma	+	120		9	(31)
36	12	24 h	AM, C, CC, H, S	+	48			(32)
69	1, 5	24 h	Eye, Plasma		24, 96			(33)
75	3	24 h	C				217	(34)
76	1, 5	24 h	Eye		96			(35)
96	3	1.5 min	AC, C, CN, H, IC, MGN			7	21	(36)
75	3	24 h	AM, C, H	+	73	7	42, 112, 175	(37)
36, 75, 116	12	24 h	BSt, C, CRB, Lungs	+	6, 24			(38)

*^a^AC, auditory cortex; AM, amygdala; BSt, brain stem; C, cortex; CBL, cerebellum; CC, corpus callosum; CN, cochlear nucleus; CRB, cerebrum; H, hippocampus; IC, inferior colliculus; MB, midbrain; Me, medulla; MGN, medial geniculare nucleus; OT, optic tract; S, striatum; TG, trigeminal ganglion; Th, thalamus; WB, whole brain*.

b *End points defined as: (1) acute: <7 days, (2) sub-acute: 7–14 days, (3) chronic: >14 days*.

#### Low-Level Blast Exposure Studies

Ahlers et al. developed a multiple blast exposure rodent model which was designed to mimic conceptually training environment of the breachers and military/law-enforcement populations exposed to multiple IEDs or breaching blast events (38). In their studies they used three BOPs: 36.6, 74.5, and 116.7 kPa (equivalent to 5.3, 10.8, and 16.9 psi) and animals were exposed 1 or 12 times with 24 h between exposures in frontal or side orientations and followed for up to 1 week. The other two shock wave characteristics, the duration, and impulse were also reported: 4.1, 4.8, and 6.8 ms, and 75.2, 175.8, and 335.5 Pa·s, respectively. To the best of our knowledge, it is one of the two published studies to date (32, 38), which used such high (12) number of exposures. Behavioral tests used were: balance beam task, passive avoidance task, evaluation of retrograde and anterograde amnesia [via passive avoidance (PA)] and Morris water maze, supplemented by immunohistochemistry analysis of amyloid precursor protein (APP). There were only transient effects of blast exposure: impairment of gross motor function in rats exposed to 116.7 kPa with recovery after 2 h and no changes after that. This effect was more severe in the side orientation, compared to animals which faced the blast wave. Exposure to BOP produced significant anterograde impairment only in the 116.7 kPa (side) and the 74.5 kPa (frontal) groups. Authors reported no accumulation of APP in white matter at any time point post-injury in the cerebrum, brainstem, and cerebellum, and there was no evidence of altered APP immunostaining within cell bodies or perivascular regions. The follow up study (32), focused on the effects associated with low-level blast exposure retained the exposure conditions from the original experimental design (BOP: 36 kPa, 12 exposures with 24 h in between) and focused on characterization of endothelial glycocalyx (a carbohydrate-rich layer that lines the luminal side of the vascular endothelium) and related behavioral tests. The multiple exposures to LLB resulted in a mild performance decrement in the MWM task, which suggests learning and memory deficits. Authors reported that: “administration of hyaluronidase, an enzyme that binds to and degrades hyaluronan (a major structural component of the glycocalyx) before blast exposure reduced the observed behavioral deficits and induced thickening of the glycocalyx layer.” The connection between the suggested BBB impairment and observed behavioral changes was not explored.

#### The Animal Model of PTSD Associated With Repeated Blast Exposure

The association of post-traumatic stress disorder (PTSD) traits in association with mild blast TBI was a subject of work of Elders and colleagues (37). Rats were exposed three times to a shock wave with 74.5 kPa (10.8 psi) BOP spanning 24 h, and then subjected to a battery of behavioral tests including locomotor activity and open field, light/dark emergence, elevated zero, Morris water, and eight-arm radial mazes, prepulse inhibition/acoustic startle, contextual and cued fear conditioning, and predator scent exposure. In this study, blast exposure induced a variety of PTSD-related behavioral traits that were present many months, including anxiety, enhanced contextual fear conditioning, and an altered response in a predator scent assay. Elevation of the stathmin-1 in the amygdala was also reported, which is implicated in the generation of fear responses. Authors concluded that due to the application of general anesthesia, blast-related mTBI in the absence of any psychological stressor, induced chronic PTSD-related traits. The follow-up work focused on remediation of the anxiety and reverse PTSD-related behaviors and characterization of chronic neurovascular disruption in Long-Evans rats using the same injury paradigm (3 × 74.5 kPa within 3 days) (29, 30). The administration of BCI-838, a group II metabotropic glutamate receptor (mGluR2/3) antagonist prodrug (its active metabolite BCI-632 has pro-neurogenic, procognitive, and antidepressant activities in animal models) reversed PTSD related behavioral impairment and improved anxiety and fear-related behaviors and long-term recognition memory. Authors reported that the treatment with BCI-838 also increased neurogenesis in the dentate gyrus (DG) of blast-exposed rats (30). The perivascular disruption was reported in animals 6 weeks post-exposure, including decreased levels GFAP, α-internexin, and several neuronal filament proteins via 2D SDS PAGE and MALDI-TOF spectrometry. These results demonstrate that multiple blast exposure at relatively low levels has the potential to disrupt gliovascular and neurovascular interactions, which was confirmed via electron microscopy (swelling and degeneration of astrocytic end-feet in the brain cortical vasculature).

The effect of antioxidant [N-acetylcysteine (NAC) and 2,4-disulfonyl α-phenyl t-butyl nitrone (HPN-07)] treatment on biomarker expression was evaluated in the brains of Long-Evans rats exposed to three successive 96.5 kPa (14 psi) BOP exposures (the time interval between exposures was 1.5 min) (36). The set of examined biomarkers included: (1) 4-hydroxy-2-nonenal (4-HNE) an oxidative stress marker, (2) c-fos: an immediate early gene, (3) glial fibrillary acidic protein (GFAP), and (4) two markers of axonal injury: APP and 68 kDa neurofilament (NF-68). The blast exposure induced acute (24 h post blast) upregulation of 4-HNE, APP and c-fos, which gradually decreased by day 7, while GFAP and NF-68 levels continued to increase for up to 21 days. The antioxidant treatment was generally an effective strategy to reduce the levels of tested biomarkers. Additionally, authors reported lack of neuronal cell death and no evidence of apoptosis via caspase-3 staining, and that the antioxidant treatment reduced blast-induced ABR (auditory brainstem response), DPOAE (distortion product otoacoustic emission) level shifts, and hair cell loss.

#### The Effect of Repeated Blast on DNA Methylation

The chronic (8 months) changes in DNA methylation were evaluated in the frontal cortex of rats exposed three times to 74.5 kPa BOP within 24 h of each other (34). Brain lysates were used to separate cells into neurons and glia, and the DNA methylation profiles in these cells were then studied using ERRBS (Expanded Reduced Representation Bisulfite Sequencing) method. It turned out neurons, and glia have distinct methylation profiles characterized by an increase in global methylation in neuronal vs. glial cells (*p* < 10^−7^). The differential (blast vs. control animals) DNA methylation perturbations were noted in blast-exposed animals within 458 and 379 genes in neurons and glia, respectively. The gene ontology (GO) and pathway analysis revealed that methylated neuronal genes showed enrichment in cell death and survival, nervous system development and function. The ERRBS gene expression analysis was validated using a set of 30 differentially methylated neuronal and glial genes selected based on GO analysis, e.g., a 1.2-fold change in gene expression of the serotonin N-acetyltransferase gene (Aanat) in blast animals was noted (*p* < 0.05). This level of change is relatively modest, and the authors noted it is presently unclear how it might affect the brain function. Interestingly, this work is the first genomic study on changes in DNA methylation induced by multiple blast overpressure exposure.

#### Ocular Trauma After Repeated Blast Exposure

The ocular trauma associated with repeated blast was also evaluated (33, 35). Rats were exposed to a single (1x) or repeated (5x) blast with 70 kPa (10 psi) average BOP with the Δt = 24 h. Activated caspase-3 was detected in ocular tissues, and it scaled with the number of exposures. GFAP and CD68 were identified in the retinas from all animals subjected to BOP, but not quantified. Single and repeated blast exposure resulted in increased expression of TRPV1 (Transient Receptor Potential Vanilloid 1), CGRP (calcitonin gene-related peptide), SP (substance P), and ET-1 (endothelin-1), but except for TRPV1, no quantification was performed on the IHC samples, and no verification with additional methods. Neutrophil infiltration was significant only in multiple exposed animals. A study utilizing the same set of exposure conditions and biomarkers supplemented with ET-A and Ca^2+^ imaging, found increased levels of TRPV1 and ET-1 in trigeminal ganglion (31).

### Sprague-Dawley Rats

#### Study Design

The majority (58% of studies, Table 2) used exposures in the BOP range falling between 130 and 140 kPa (40–42, 45, 46, 48, 49), while the remaining 42% of studies included a blast exposure below 100 kPa (39, 42, 44, 47, 50).

**Table 2:** The summary of experimental variables (exposure conditions: BOP, number of exposures and inter-exposure interval, analyzed brain region, behavioral testing and end points) in studies using Sprague Dawley rats.

**BOP, kPa**	**Number of exposures**	**Inter-exposure interval**	**Brain region (other tissue or organ)^**a**^**	**Behavior**	**End points**^**a**^			**References**
					**Acute, hours**	**Sub-acute, days**	**Chronic, days**	
89, 552	1, 2	30 min	BSt		24			(39)
106, 134, 123	3	30 min	CRB	+		7	90	(40)
131	2	1 min	C		24		28	(41)
83, 131	2	1 min	BSt, CBL, OT	+	1, 6, 24	7		(42)
110	3	30 min	C, H		24			(43)
69, 110	1, 3	30 min, 24 h	CRB		24	5		(44)
126	2, 3	1 min	CRB		72			(45)
138	1, 5	24 h	CBL, H, Th				42	(46)
75	3	24 h	–	+			56	(47)
138	1, 5	24 h	Plasma				42	(48)
138	1, 5	24 h	AM, C, H, Plasma		1, 2		16, 22	(49)
62	1, 2, 3	1 min	Lungs		1, 6, 24			(50)

a *See Table 1 annotations for details*.

The number of exposures ranged from 1 to 5 (Table 2), and a single exposure was used as a reference in 50% of studies. The most common exposure numbers were two and three exposures (42%, in both cases, respectively). The remaining three studies had an injured group with five total exposures. Elsayed and Gorbunov's (50) paper was the only investigation which varied the number of multiple exposures, including injury test groups with one, two, and three exposures.

The inter-injury interval also varied among the studies examined. Four studies exposed their animals successively, where the animal was kept under anesthesia for the duration of testing, and there were <3 min between each exposure (41, 42, 45, 50). It constituted the most severe blast exposures investigated and were argued to be analogous to same-day exposures in humans. Four studies allowed for 30 min between exposures (39, 40, 43, 44) and five studies examined the effect of daily exposures (44, 46–49). These allowed for the animal to come out of anesthesia between subsequent blasts. Kawoos et al. (44) examined the effect of two different inter-exposure intervals, comparing the injury response between three 110 kPa exposures with 30 min and 24 h between exposures.

One study sought to compare results from exposure in the WRAIR shock tube with a blast-driven shock tube design (400 mm diameter, 1.5 m long), generating an overpressure using an explosion of Swedish Army plastic explosives (39). It resulted in a vastly different incident shock wave, with the blast-driven shock tube generating an 800 kPa overpressure, 0.25 ms duration, incident wave. It is a substantially higher overpressure and lower duration than those waveforms generated by the WRAIR shock tube (110 ± 26.3 kPa overpressure, 8.47 ± 1.50 ms duration).

#### Physiological Measurements and Monitoring

Physiological health is vital to assess the effect of shock exposure on specimen health. Longitudinal monitoring of physiological measurements was conducted to evaluate the long-term changes that occur following a single and multiple shock exposures. It was found that there were no significant differences observed in pulse distension or breath rate for single or multiple exposure animals when examined longitudinally for 42 days (one vs. five daily 138 kPa exposures). Transient significant differences were observed in arterial oxygenation levels and heart rate between injured groups and sham animals, while no significance was observed for the number of exposures (48).

Elevated levels of intracranial pressure (ICP) could signify changes which, unchecked, could contribute to adverse outcomes, e.g., increased mortality, following TBI (44). ICP reportedly exhibits a transient or sustained increase after single and multiple shock exposures, showing dependence on blast overpressure, number of exposures, and Δt (44). A single low-level shock (72 kPa) produced a transient response, with ICP returning to baseline within 3 h, while multiple exposures (three exposures every 30 min) produced a sustained increased ICP which lasted up to 5 days after injury. Higher BOPs (72 vs. 110 kPa) produced a biphasic response, with an immediate increase in ICP after exposure and a gradual increase to the maximum ICP, occurring 1–2 days after exposure. When comparing the biphasic responses of the ICP after single and multiple shocks (1 vs. 3), multiple shocks induced a more rapid increase in ICP, with the peak occurring more quickly. Increasing the Δt (30 min vs. 24 h) lowered the peak ICP and eliminated the biphasic response, with ICP remaining elevated (44). Results indicate that ICP elevation shows dependency on the number, intensity, and time between exposures. It supports a hypothesis that rbTBI outcomes will differ from a single exposure and that the time between exposures and the number of exposures exacerbates is important when considering outcomes.

#### Pathological Changes

The assessment of gross pathology and morphological changes of the tissues can be used to assess the injury. Only one study noted the presence of gross pathological changes, i.e., contusions in the cortical surface 3 days following two subsequent 130 kPa exposures (45). Elsayed and Gorbunov performed extensive pathological investigations, yet only presented the histological examination of the lung tissues. Brain tissue was reported to be less sensitive to shock exposures when compared with lung tissue, but no quantification was presented (50).

Some groups used histopathological examinations to stain for signs of cellular damage. The hematoxylin-eosin staining 1 day after three 110 kPa exposures with 30 min between exposures. The cortex showed signs of cytoplasmic vacuolization, nucleus shrinkage, and perivascular vacuolization (43). The hippocampus showed eosinophilia of the cytoplasm in CA1, CA2, and CA3, but not in the dentate gyrus (43). Silver staining was used by several groups to grade neurodegeneration and to examine patterns of morphological changes. Three days after two consecutive 130 kPa exposures, silver staining revealed mild to moderate neuronal degeneration in the multiple exposures experimental group in the cortex (45). Additionally, patterns of diffuse axonal injury (DAI) were observed following single and multiple exposures in the cerebellar white matter, optic tracts, and the cerebral peduncles, with double-exposed groups, exhibited a stronger response (45). With the same exposure conditions, Wang et al. reported that axonal damage in the cerebellar white matter persists in both injury groups for 7 days and the double-exposed group continues to show the highest levels of axonal injury (42). Kawa et al. used Fluoro-Jade and beta-amyloid precursor protein staining to label degenerated neurons in the hippocampus 1 day after one and two exposures spaced by 30 min in two shock conditions, one high BOP-low impulse exposure condition in the KI shock tube (800 kPa, 0.25 ms duration) and one low BOP-high impulse exposure condition in the WRAIR shock tube (89 kPa, 8.5 ms duration). No positively stained neurons were identified, implying that neither exposure condition induced neurodegeneration. DNA fragmentation and presumed apoptosis were examined in the hippocampus 2 h and 22 days after single and multiple shock exposures using terminal deocynucleotidyl transferase dUTP nick end labeling (TUNEL). Notably, no difference was observed between single and multiple exposures (five daily 138 kPa exposures) and the significant changes were only seen in the granular cell layer of the ventral hippocampus of the multiple exposed group at the chronic time point, at 22 days post-injury.

Several studies used MRI diffusion tensor imaging (DTI) to quantify changes to the white matter tracts following single and multiple shock exposures. Different quantitative measures, including fractional anisotropy (FA), radial diffusivity (RD), axial diffusivity (AD), and apparent diffusion coefficients (ADC), were compared between experimental groups via pair comparisons at the voxel, white matter tract, or regions of interest (ROI) levels. Comparion of a single exposure to five daily mild shock exposures, a significant difference in AD and RD at the thalamus and cerebellum was observed at 2 h after injury (46). The decreased FA and increased RD in the cerebellum was also seen 72 h after two consecutive exposures when compared to a single exposure and an uninjured sham (45). At 7 days post-injury, widespread changes in AD, RD, and AFD, occurred in the thalamus, cerebellum, corpus callosum, and axial hindbrain, while no significant changes in FA were found (40).

Examination of chronic timepoints results in less cohesive observations. At 42 days, no significant changes are reported when comparing one and five daily 137 kPa exposures (46). However, at 90 days three exposures (107, 133, and 123 kPa) delivered every 30 min resulted in widespread changes in FA, AD, RD, and ADC using both voxel-wise and ROI comparisons (40). These seemingly contradictory results could highlight the importance of the inter-exposure interval in the development of chronic pathologies. Daily exposures did not develop a chronic response. The lack of significant evidence of cumulative effects was seen in two other studies which used daily exposures, where no differences were observed between the single exposure and the multiple exposure models (44, 48). It could highlight that daily exposures allow ample time to recover between exposures (49).

#### Biochemical Changes

Neuronal loss following rbTBI was implicated through immunohistochemical evaluation of neuronal nuclei (NeuN) showed a significant decrease in the immunoreactivity of neurons in the cortex and hippocampus 1 day after three 30 min 110 kPa exposures (43). Further neuronal damage was evaluated by examining plasma concentrations of neurofilament-H (NF-H) and neuron-specific enolase (NSE) following five daily 138 kPa exposures. NF-H plasma concentrations were elevated compared to sham 2 h after exposure and both NF-H and NSE concentrations were elevated 22 days post-injury, but no differences were observed between single and multiple exposure experimental groups (49). Under the same exposure conditions, NF-H, and myelin basic protein (MBP) plasma concentrations are elevated in injury groups, with the rbTBI group exhibiting a higher MBP concentration when compared to the single exposure group (48). Additionally, increased immunofluorescence of doublecortin (DCX) in the hippocampus 22 days after injury indicates a late onset of *de novo* neurogenesis (49). In the same study the protein levels in tissue homogenates that indicate neuron functionality were also investigated. Tau protein concentration increased following a single 138 kPa exposure in the hippocampus 22 days post-injury when no change was seen 2 h after injury or following five daily 138 kPa exposures (49). The rbTBI group exhibited a higher concentration of n-cadherin (NCad) in tissue homogenates of the prefrontal cortex 22 days post-injury (49).

Reactive astrogliosis following injury was probed by evaluating protein levels of glial fibrillary acidic protein (GFAP) within the brain tissue or plasma. An increase in GFAP immunoreactivity 2 h after one 138 kPa exposure in the hippocampus was noted (49). At 22 days, the rbTBI group, with five daily 138 kPa exposures, showed more immunoreactivity than the single exposure group (49). Plasma concentrations of GFAP were elevated for both the single and multiple exposed groups, but no difference was seen between the two injury conditions (49). Ahmed et al. reported an upregulation of plasma GFAP 42 days post-injury for a single and five daily 138 kPa exposures, with the rbTBI exhibiting higher plasma concentrations when compared to the single exposure (48).

A proposed injury mechanism following TBI is the development of neuroinflammation mediated by blood-brain barrier damage (51, 52). Kamnaksh et al. and Ahmed et al. examined concentrations of von Willebrand factor (vWF), a protein involved in mediating inflammation, following one or five daily 138 kPa exposures. No changes were seen in concentrations of vWF in tissue homogenates of the amygdala, hippocampus, and prefrontal cortex 2 h and 22 days post-injury in either the single or multiple exposed groups (49). However, by 42 days, significant increases in the plasma concentrations of vWF, matrix metalloproteinase 8 (MMP8), formyl peptide receptor 1 (FPR1), and p38 mitogen-activated protein kinase (p38) were observed for both injury groups (48). No changes were seen in the levels of toll-like receptor 9 (TLR9), and the plasma concentrations of chemokine (C-C motif) receptor 5 (CCR5) were elevated in the single exposure group alone (48). Chemokine (C-C motif) ligand 2 (CCL2) concentrations in CSF, plasma, and tissue homogenates highlighted that CCL2 is elevated after two consecutive 130 kPa exposures (6, 24 h, and 7 days post-injury) (42). Additionally, total RNA extracted from the cerebellum showed increased CCL2 gene expression and CCL2 mRNA as well (42).

Oxidative stress has also been identified as a secondary injury mechanism following rbTBI. Gu et al. examined 3-nitrotyrosine (3-NT), a marker of oxidative/nitrosative damage, following three 30 min 110 kPa exposures. 3-NT immunoreactivity was increased in the cortex 1 day after injury, but no difference was observed in the hippocampus. When compared to sham, tissue homogenates of the cortex at 1 and 28 days post-injury, also showed a higher total ROS level following two consecutive 130 kPa exposures (41). Untargeted metabolomics at 28 days post-injury highlighted that methionine and ascorbic acid metabolism were significantly altered after the blast. Methionine metabolism disruption was seen via changes in cysteine, methionine sulfoxide, and glutathione (41). Likewise, ascorbic acid metabolism showed a persistent oxidative stress for up to 28 days post-injury, with decreased levels of ascorbic acid and increased levels of dehydroascorbic acid and its metabolic derivatives (41). Proteomics analysis of plasma 42 days after one and five daily 138 kPa exposures highlighted increased concentrations of 4-hydoxynonenal, hypoxia-inducible factor 1α, and ceruloplasmin. For all three proteins, concentrations were higher in the rbTBI experimental group.

Several groups investigated markers of vascular health following single and multiple exposures. Immunoreactivity of endothelin-1 receptor A (ETRA) and aquaporin-4 (AQP-4) were notably higher in the cortex and hippocampus 1 day following three 30 min 110 kPa exposures with respect to sham (43). Changes to plasma concentration VEGF were observed following one or five daily 138 kPa exposures. VEGF concentrations were elevated for both single and multiple exposure groups 2 h post-injury (49), in single exposures 22 days post-injury (49), and in both single and multiple exposure groups 42 days post-injury (48). The lack of significance seen at 22 days post-injury can be attributed to inadequate experimental group size (*n* = 7), as VEGF plasma concentration in the multiple exposure group at 22 days was elevated when compared to a single injury, and the multiple exposed sham group. Kamnaksh et al. (49) also reported that fetal liver kinase 1 (FLK-1) levels were unchanged and comparable to sham values in the amygdala, hippocampus, and prefrontal cortex at 2 h and 22 days after one and five daily 138 kPa exposures (49).

The *in situ* hybridization was used to probe the mRNA levels for galanin, tyrosine hydroxylase (TH), and tryptophan hydroxylase (TPH2) in the brainstem (39). The purpose of this analysis was to compare injury modalities resulting from exposures with a Δt = 30 min in two rbTBI models conditions, one high BOP-low impulse exposure condition in the KI shock tube (800 kPa, 0.25 ms duration) and one low BOP-high impulse exposure condition in the WRAIR shock tube (89 kPa, 8.5 ms duration). Transcript levels showed dependence on exposure type and animal handling (39). Increases in TH and galanin were significant in the KI experimental model, but significance was not observed in the WRAIR model and, importantly, no effect was observed when comparing single and multiple exposures (39).

The existing results highlight that consistent exposure conditions across various exposure models facilitate the direct comparison of experimental findings. However, there exists a perpetuation of animal group sizes and exposure conditions which do not show significant differences between single and multiple exposure groups. A third of the studies with SD rats do not include an experimental group with a single exposure, and another 33% cite that no cumulative effect was observed, with results trending toward significance. In general, the disparity between selected endpoints and inter-exposure intervals were not ideal for yielding significant results and can be regarded as aggravating factors preventing direct comparison between adopted injury models. Additionally, there were no investigations of the effect of BOP or Δt on the biochemical, histological, or immunohistochemical changes following rbTBI.

#### Behavioral Alteration

Behavioral assessments were conducted in 25% of studies which involve Sprague-Dawley rats. The goal of behavioral assessments is to connect exposure conditions to neurobehavioral changes which may mirror symptomology seen in humans. Studies in SD rats examined behaviors associated with PTSD, spatial learning, spatial memory, anxiety, depression, and motor coordination.

To evaluate the connection between rbTBI and PTSD-like symptoms, Genovese et al. investigated the conditioned fear response following three daily 74.5 kPa exposures. Fear conditioning was cultivated via an inescapable electric shock (IES), coupled with an audio-visual stimulus. Longitudinal testing for 2 months following repeated shock exposures revealed, counter-intuitively, that coupling a rbTBI exposure with an IES reduced the conditioned fear when compared to a sham animals (47). It was concluded that this result can be attributed to a reduction in inhibitory function after rbTBI (47).

Spatial learning and memory were evaluated using the Barnes maze, in which a specimen is placed on a circular platform. An escape box is located under one of twenty holes evenly spaced around the circumference of the platform. Visual cues around the room and pre-training the animal facilitates learning the location of the escape box. The latency to find the escape box over the course of 5 testing days following single and multiple exposures (Δt = 24 h, BOP = 138 kPa exposures). In the acute phase, no significant differences were seen between experimental groups (49). A trend was observed at later time points (17–21 days post-injury), where multiple exposed animals (*n* = 7) tended to have a longer latency time than the multiple sham group (*n* = 6), however the specimen number was not high enough to see significance (49).

Anxiety- and depression-like behaviors were evaluated using the open field test, where a specimen is placed in a large plexiglass arena and the horizontal activity, vertical activity, and time spent in the center of the field are tracked over the course of the testing session. Changes in the vertical activity of a specimen indicate alterations in exploratory behavior and act as an index of depression-related behaviors (49). Directly after injury (24 h), multiple exposed animals (five daily 138 kPa exposures) had lower vertical activity than single exposed, sham, and naïve test groups (49). No differences were seen at day 16, indicating a transient response. A single exposed animals, did not show any significant changes in vertical activity. Anxiety-like behaviors, as defined by decreased time spent in the center of the field, were seen in multiple injured and multiple sham animals 1 day after the injury, indicating that anxiety in rbTBI animals may be linked to multiple bouts of anesthesia. Further work is required to determine if the injury itself induces anxiety-like behaviors. This highlights the necessity of the inclusion of multiple sham testing groups in experimental design.

Impairments in general locomotion have been evaluated in two reports. Transient response was seen, with multiple exposed animals (five daily 138 kPa exposures) showed signs of locomotor impairment directly after exposure, but no difference was seen at day 16 (49). Similarly, two exposures increased motor impairment in a rodent model, as defined by the rotarod test. The rotarod is a test which quantifies changes in motor coordination and balance through a forced motor activity which can be longitudinally compared to baseline performance. Briefly, a specimen is placed on a rotating drum and the latency to fall is recorded. Following a single shock exposure, motor impairment and coordination were not altered (42). However, significantly higher motor impairment was observed when animals were exposed to two consecutive shocks, with significant differences between single and double exposed injury experimental groups (42). These studies indicate that a single exposure is not enough to affect the motor coordination, but cumulative exposures may impair locomotion.

#### Treatments

Two treatments have been attempted to mitigate the effects of multiple shock exposures in the SD animal model, and both were focused on the ICP. Mechanical compression of the internal jugular vein (IJV) was performed prior to shock exposure to determine if an increase in ICP and/or an increase in brain volume could attenuate TBI following repeated exposures. The hippocampal NeuN and AQP-4 levels showed that IJV compression was partially effective in prevention of neuronal loss, edema formation, and vascular dysfunction. Although treated groups did not return to baseline values, results indicate that increased ICP and/or increased brain volume could have a neuroprotective effect (43). Kawoos et al. tested the effect of intraperitoneal injections of *N*-acetylcysteine amide (NACA) on the ICP in the acute and sub-acute period after injury. This antioxidant was found to effectively reduce blast-induced ICP elevation after single and multiple exposures (44).

### Studies Using C57BL/6J Mouse Model

In addition to two different strains of rats discussed earlier, a few studies have employed an inbred C57BL/6J mouse strain to investigate various physiological, pathological and neurobehavioral alterations following rbTBI (Table 3). Notably, except for one study (26), all the studies were performed in the shock tube located in Walter Reed Army Institute of Research (WRAIR). This model used 3 exposures separated by 1 and 30 min, and relatively high BOPs in the 142–144 kPa range. Majority of the studies focused on the characterization of acute effects, typically with several endpoints in the initial 24 h post-injury (Table 3).

**Table 3:** The summary of experimental variables (exposure conditions: BOP, number of exposures and inter-exposure interval, analyzed brain region, behavioral testing and end points) in studies using C57BL/6J mice.

**BOP, kPa**	**Number of exposures**	**Inter-exposure interval**	**Brain region (other tissue or organ)^**a**^**	**Behavior**	**End points**^**a**^			**References**
					**Acute, hours**	**Sub-acute, days**	**Chronic, days**	
131	1, 3	24 h	CBL	+	1, 4, 14, 30		120	(26)
92	3	1 + 30^b^	C, CBL	+	2, 6, 24	7		(53)
142	3	1 + 30	C, CBL		6, 24, 72	7	14	(54)
142	3	1 + 30	C, CBL, H, MB		3, 6, 24, 72	7	14	(55)
145	1, 3	1 + 30	C		1, 6, 24			(56)
142	3	1 + 30	C, Plasma		2, 24, 72			(57)
145	3	1 + 30	CBL, Plasma		6, 24			(58)
145 (69, 103)	3	1 + 30	Plasma Liver, Muscle		1, 6, 24			(59)
142	3	1 + 30	C, CBL, H, MB		6, 24			(60)
142	3	1 + 30	CBL, Inner ear		6, 24			(61)
142	3	1 + 30	C, CBL, H, MB, Me		3, 6, 24, 72	7	14	(62)
142 (96, 172)	3	1 + 30	C, CBL	+	2, 4, 6, 24, 48, 72, 120			(63)

a *See Table 1 annotations for details*.

*^b^1 + 30: animals were exposed three times with inter exposure interval of 1 min. between exposures 1 and 2, and inter-exposure interval of 30 min. between exposures 2 and 3*.

#### Biochemical Markers

Wang et al. (63) exposed 8–10 weeks old C57BL/6J male mice weighing 22–26 g. After an initial titration of BOPs at 96, 142, and 172 kPa, a BOP of 142 kPa was selected as primary BOP intensity. Assessment of mortality rates in the animals revealed that at 96 kPa (13.9 psi), there was no mortality observed whereas a single exposure at 142 kPa resulted in 8% mortality, which further increased to 28% after 3 repeated exposures at the same BOP. These observations clearly indicate that there was a cumulative impact of shockwave load. Similarly, neurological scores (righting reflex) worsened in repeated compared to single exposure groups. A significantly higher loss of body weights was also observed in animals exposed to multiple BOPs. Neuropathological observations revealed a significant increase in brain water content (edema) within 4 h in animals exposed to multiple blasts, however by 24 and 48 after injury, the water content restored to that of normal. Multiple blast injury also displayed increased oxidative stress as indicated by a progressive crease in ROS production between 4 and 48 h in brain homogenates and such increased ROS correlated with the increased neurodegeneration as indicated by significantly increasing the number of FluoroJade-positive neurons. These neuropathological alterations also disturbed motor performance. It is noteworthy that a single blast exposure itself significantly decreased the latency to fall in a rotarod device. However, such defects were reversed in animals exposed to a single blast after 5 days, whereas multiple exposure group continued to display motor deficits. In another study by the same group (53), much lower BOP (92 kPa) was employed. A progressive DNA damage was found with an incremental increase in fragmentation of DNA in cerebral cortex and cerebellum and a concomitant rise in cell-free DNA levels in plasma indicating cerebral and systemic cellular DNA damage. Additionally, a profound increase in mitochondrial damage was also reported as estimated by decreased mitochondrial membrane potential loss and increased release of cytochrome C into the cytosol in animals exposed to multiple blasts. It indicates that shockwaves not only disrupt the cellular architecture but also damage sub-cellular structures. Additional studies by lead by Peethambaran (56), showed a significant reduction in ATP levels in the cerebral cortex of animals exposed to both single or rbTBI at 145 kPa 24 h post-injury. An examination of possible reasons for reduced brain ATP by proteomic analysis further revealed a reduction in pyruvate dehydrogenase (PDH) and mitochondrial glutamate-oxaloacetate transaminase (mGOT) which indicate defective glycolysis and mitochondrial tricarboxylic acid (TCA) cycle enzyme pathways contributing to reduced ATP levels following repeated blast exposure.

Few follow up studies have also been performed using mice model of repeated and single blast exposure to identify biomarkers of injury. Accordingly, studies by Peethambaran et al. (61) subjected mice to single exposure at 70, 103, and 145 kPa, as well as rbTBI at 145 kPa repeated 3 times. A significant increase in plasma levels of aspartate aminotransferase (AST), alanine aminotransferase (ALT) creatine kinase (CK) and lactate dehydrogenase enzymes (LDH) were observed. All the enzyme levels were significantly altered in mice exposed to single BOP, and there was a progressive increase in the levels of these enzymes as a function of the BOP, observed as early as 1 h post injury. Multiple exposures at 145 kPa significantly increased the plasma levels of these enzymes as early as 1 h after the blast and continued to show increased levels until 24 h. Interestingly, mice covered with body armor did not show any changes in the plasma levels of these enzymes indicating that increased plasma levels of these enzymes were due to damage to systemic organs and that body armor offered an effective protection. Considering all enzymes studied are markers of liver damage, a follow up histopathological studies demonstrated moderate coagulative necrosis, pyknosis, and karyolysis. Additional studies performed by the same investigators (57) report increase plasma levels of serotonin and myeloperoxidase which indicate that repeated blast exposure also causes hematological changes including platelet aggregation and polymorphonuclear changes. These studies have also identified simultaneous changes in brain histopathology in cerebral cortex showing a prominent cerebral vasoconstriction.

Increased plasma levels of glial fibrillary acidic protein (GFAP) and Tau proteins were identified in a model of rbTBI where 145 kPa BOP was used (58). The degradation of cytoskeletal marker α-spectrin was also observed indicating disruption of cellular integrity in the brain (54).

#### Hearing Impairment

Mice models have also been used to identify hearing impairments following repeated blast exposure considering hearing loss and development of tinnitus are most prevalent in service members (64, 65). Accordingly, proteomic studies were performed using inner ear and cochlea, as well as brain tissue homogenates of mice exposed to 145 kPa BOP for 3 times, to identify changes in hearing-related proteins. The increased protein levels of calretinin, a calcium binding protein, and parvalbumin, a marker of neurons in both cochlea, and cerebellum were demonstrated. The calretinin and parvalbumin alterations are associated with hearing impairment (66, 67) and changes in these proteins following repeated exposure strongly suggest that rbTBI can cause hearing impairment. An additional study by the same group also observed differential expression of auditory-related genes such as otoancorin, otoferlin and protocatherin-α in the hippocampus of mice exposed to rbTBI at 145 kPa (21 psi) with Δt = 3 (60).

#### Neurotransmitters

Alterations in specific neurotransmitter's enzymatic systems following repeated blast exposure were also reported. Acetylcholine is a neurotransmitter and neuromodulator involved in neuromuscular coordination. Acetylcholine esterase (AChE) is a central part of cholinergic neurotransmission as this enzyme catalyzes the hydrolysis of acetylcholine (68). Few studies reported a differential alteration in the activity of AChE as a function of time and such changes were found to be region-specific in the brains of mice exposed to 145 kPa BOP repeated 3 times. The increase in AChE in frontal cortex 6 h post injury was noted, whereas its levels significantly decreased in hind cortex and hippocampus at 6, 24 h, and 3 days post injury. Cerebellum showed a biphasic response: initial decrease at 6 h followed by increase at 3 days post-injury. Analysis of blood levels of AChE showed a decrease at 6 and 24 h, and 3 days post-injury indicating a differential response of this enzyme to rbTBI. Such organ- and tissue-specific temporal variations in may in part be due to differences in the density of cholinergic neurons in different brain regions so that the ACh metabolism may be modulated accordingly.

#### Correlation With Human Brain Pathology

A comprehensive study performed by research group at the University of Washington (26), adopted different mouse injury model than other reports included in this review (Table 3). In this model mice were exposed 131 kPa BOP repeated 3 times with 24 h delay between consecutive exposures. They performed a variety of pathological and behavioral studies with a focus only on the cerebellum. Both single and repeated blast exposures at 131 kPa caused disruption of blood-brain barrier (BBB) as indicated by extravasation of fluorescent dextran (10 kDa) into the ventral region of cerebellar parenchyma. Analysis of cerebellar Purkinje cell number showed a moderate loss of cells in animals exposed to a single blast, whereas multiple exposures synergistically increased the Purkinje cell loss. A robust number of activated microglial cells was observed in the vicinity of vascular rupture in the cerebellum as indicated by increased expression of Iba1. Further, microglia in the vicinity of vascular rupture appear more activated than their distant counterparts as indicated by more pronounced colocalization of Iba1 with CD68 (a marker of activated microglia) immunofluorescence. Both BBB disruption and microglial activation in cerebellum were concomitant with motor deficits, synaptic (loss of PSD95) and axonal pathology and reactive gliosis.

## Conclusions

Three strains of rodents are currently employed to investigate mechanisms of repeated exposure to blast overpressure using shock tubes: C57BL/6J mice, Sprague-Dawley, and Long-Evans rats. The character of exposure conditions, i.e., the blast intensity levels, and inter-exposure intervals, employed in these studies suggests that they were developed to replicate etiology of mild blast TBI of improvised explosive devices (IEDs) and high-explosive detonations rather than exposure to low-level blast encountered by military and law enforcement populations in training.

Our analysis indicated existing trends in the experimental animal models developed to replicate injury conditions associated with repeated blast exposure. There seem to exist a disparity in exposure conditions between laboratories using different animal models: studies which employed Long Evans rats used Δt of 24 h and relatively low intensity BOPs (<76 kPa), while in studies employing C57BL/6J mice a relatively short Δt's (1 and 30 min) and high BOPs (~145 kPa) were used. Studies using Long Evans rats focused predominantly on the evaluation of chronic effects, while research performed on mice was focused on acute effects. Studies performed with Sprague Dawley rats are characterized by the largest diversity in exposure conditions and end point selection. However, in none of the existing studies to date a systematic evaluation of the inter-exposure interval was performed. Moreover, a viable experimental animal model replicating appropriate occupational low-level blast injury conditions must rely on available human data. Notably, peak overpressures of <70 kPa (10 psi) should be employed, and the number of exposures increased, which should be accompanied by a shortening of the inter-exposure intervals.

The discrepancies in the existing rodent models make difficult to draw general conclusions regarding existing mechanisms common for divergent injury paradigms. However, despite the scarcity of the research in this area, a number of acute and chronic mechanisms was successfully identified. There exists strong evidence that exposure to repeated blast causes transient ocular injury and hearing loss. The repeated exposure causes changes in DNA methylation, BBB impairment accompanied by microglial activation and prolonged inflammatory response. These changes translate to behavioral effects, most notably a well-demonstrated brain pathology with PTSD.

## Author Contributions

MS and MT performed the analysis and literature search. MS, MT, KR, and NC wrote the manuscript.

### Conflict of Interest

The authors declare that the research was conducted in the absence of any commercial or financial relationships that could be construed as a potential conflict of interest.
